# Supplementary material for: Effects of tai chi on postural balance and quality of life among the elderly with gait disorders: A systematic review
Source: PLoS One. 2023 Sep 28;18(9):e0287035. doi: 10.1371/journal.pone.0287035 (PMC10538728; doi:10.1371/journal.pone.0287035)
Supplement: S1 Table — (DOCX) [file pone.0287035.s001.docx]

| Keywords | The keywords were “Tai Ji” “Tai Chi” AND “Postural Balance*” “Postural Control*” AND “Gait disorders” “Locomotion Disorder*” “Ambulation Disorder*” “Gait Dysfunction*” “Unsteady Gait” AND “old people” OR “elderly” OR “senior*” OR “old adult*” OR “aged” OR “older people” OR “older adults” OR “geriatric” | |
| --- | --- | --- |
| **Database** | **Strategy** | **Results** |
| PubMed  （Date of the last search: 5.19.2023） | Search: **((((Tai Ji) OR (Tai Chi)) AND ((Postural Balance*) OR (Postural Control*))) AND (((((Gait disorders) OR (Locomotion Disorder*)) OR (Ambulation Disorder*)) OR (Gait Dysfunction*)) OR (UnsteadyGait))) AND ((((((((old people) OR (elderly)) OR (senior*)) OR (old adult*)) OR (aged)) OR (older people)) OR (older adults)) OR (geriatric))** | 21 |
| SCOPUS  (5.19.2023) | ( ( TITLE-ABS-KEY ( tai AND ji ) OR TITLE-ABS-KEY ( tai AND chi ) ) ) AND ( ( TITLE-ABS-KEY ( postural balance* ) OR TITLE-ABS-KEY ( postural control* ) ) ) AND ( ( TITLE-ABS-KEY ( gait AND disorders ) OR TITLE-ABS-KEY ( locomotion AND disorder* ) OR TITLE-ABS-KEY ( ambulation AND disorder* ) OR TITLE-ABS-KEY ( gait AND dysfunction* ) OR TITLE-ABS-KEY ( unsteady AND gait ) ) ) AND ( ( TITLE-ABS-KEY ( old AND people ) OR TITLE-ABS-KEY ( elderly ) OR TITLE-ABS-KEY ( senior* ) OR TITLE-ABS-KEY ( old AND adult* ) OR TITLE-ABS-KEY ( aged ) OR TITLE-ABS-KEY ( older AND people ) OR TITLE-ABS-KEY ( older AND adults ) OR TITLE-ABS-KEY ( geriatric ) ) ) AND ( EXCLUDE ( DOCTYPE , "re" ) OR EXCLUDE ( DOCTYPE , "cp" ) ) | 18 |
| EBSCOhost SportDiscus  (5.19.2023) | TX (Tai Ji OR Tai Chi) AND TX (Postural Balance* OR Postural Control*) AND TX ( Gait disorders OR Locomotion Disorder* OR Ambulation Disorder* OR Gait Dysfunction* OR UnsteadyGait ) AND TX ( old people OR elderly OR senior* OR old adult* OR aged OR older people OR older adults OR geriatric )  Search Options  Expanders - Also search within the full text of the articles; Apply equivalent subjects  Search modes - Find all my search terms | 314 |
| Web of Science (5.19.2023) | 1 (TS=(Tai Ji)) OR TS=(Tai Chi)  2 (TS=(Postural Balance*)) OR TS=(Postural Control*)  3 ((((TS=(Gait disorders)) OR TS=(Locomotion Disorder*)) OR TS=(Ambulation Disorder*)) OR TS=(Gait Dysfunction*)) OR TS=(Unsteady Gait)  4 (((((((TS=(old people)) OR TS=(elderly)) OR TS=(senior*)) OR TS=(old adult*)) OR TS=(aged)) OR TS=(older people)) OR TS=(older adults)) OR TS=(geriatric)  5 #1 AND #2 AND #3 AND #4  By advanced search, in order.，search step by step.  Copy query link：  <https://www.webofscience.com/wos/alldb/summary/c2d23581-ec3a-4461-b958-f237a88fbd5c-795744a6/date-descending/1>  <https://www.webofscience.com/wos/alldb/summary/64624c73-37fd-4ff0-8b0d-de2b2480b406-7956a77a/date-descending/1>  <https://www.webofscience.com/wos/alldb/summary/5841677a-b1f1-414e-a562-a2c4d2d777e4-7956befc/date-descending/1>  <https://www.webofscience.com/wos/alldb/summary/29b231d7-cd76-4481-ab0c-0187b0bd56f2-7956e9d8/date-descending/1>  https://www.webofscience.com/wos/alldb/summary/89defb96-f7ae-4671-80c5-056f970d359-795719c1/date-descending/1 | 45 |
| CNKI  (5.19.2023) | 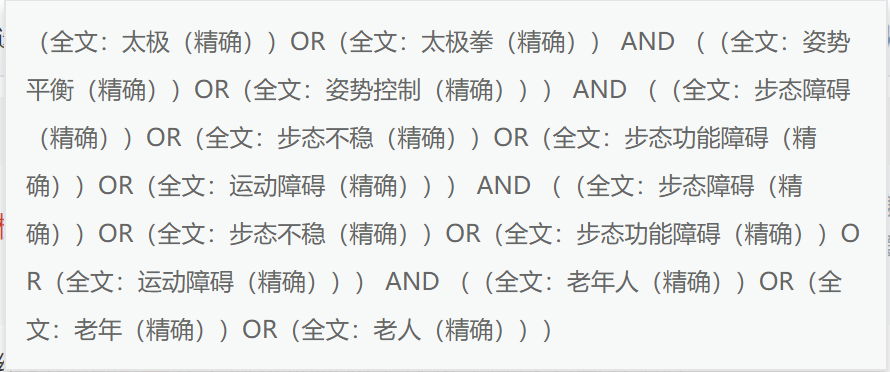 | 174 |
| Google Scholar  (5.19.2023) | Tai Ji” “Tai Chi” AND “Postural Balance*” “Postural Control*” AND“Gait disorders”“Locomotion Disorder*”“Ambulation Disorder*”“Gait Dysfunction*”“Unsteady Gait”AND “old people” OR “elderly” OR “senior*”  Find articles that contain all words | 9 |
| Total |  | 581 |
| Final Results |  | 16 |
