# Supplementary material for: Effects of tai chi on postural balance and quality of life among the elderly with gait disorders: A systematic review
Source: PLoS One. 2023 Sep 28;18(9):e0287035. doi: 10.1371/journal.pone.0287035 (PMC10538728; doi:10.1371/journal.pone.0287035)
Supplement: Attachment — Submitted filename: Response to Reviewers.docx [file pone.0287035.s003.docx]

Many thanks to the reviewer for his suggestion on manuscript number PONE-D-23-09135. Based on these comments, we have carefully revised the manuscript.

Dear Editor

We greatly appreciate your and the other reviewers' thoughtful comments. Based on these comments and suggestions, we have carefully revised the manuscript. All changes to the text are displayed in blue. We hope that new manuscripts will meet the standards of your journal.

Below you will find our point-by-point responses to the reviewers' comments/questions:

Reviewer #1:

1. for example some words like elderly and older adults have been quoted differently, I suggest you include just older adults and delete elderly.  gait and balance problems can be gait and balance impairments.  line 50 and 60 and other words.

**Response:** elderly has been changed to older adults (Lines 13, 14, 16, 41, 59, 60, 61, 68, 76, 79, 270, 322.), and gait and balance problems have been changed to gait and balance impairments (Lines 237, 240).

line 50 has been changed from Chinese martial arts to Chinese martial arts. line 60, change elderly to older adults.

2. line 21-24 Some confusion, difficult to understand, you need to add population, and then factors and measures

**Response:** Tai Chi intervention can affect populations with Parkinson's disease, no movement, mild cognitive impairment, chronic stroke, sedentary, fear of falling, or history of falling. Postural instability is associated with balance, gait, UPDRS III, mobility, leg strength, and falls. Only two articles looked at quality of life.

3. line 30: reduce falls . Did you have the numer of fallers and no fallers, so you cannot say reduce or prevent falls you used measues to decrease the risk of falls

**Response:** Line 30 is deleted.

4. line 45-46: exercise is non drug ( no understand) and all patients (which patients)

**Response:** Lines 45-46 non drug changed to complementary treatment.

which patients changed: for example, for some older adults, many exercise activities are either too intense or too monotonous, which makes them unable to persist.

5. Outcomes: Balance, mobility, gait ability, strength, fall rate, quality of life (QoL)
   You need to explain better the outcomes, which measurements each one were used

as you mentioned before postural balance is different than balance

fall rate this is the number of falls or fallers and no fallers and you mentioned fear of falling that is different

**Response:** Outcomes:

1. balance: Lines 184-185 regarding TUG deleted.

Lines 185-186 were changed to the single-leg stance test was reported in three studies on postural balance

Line 187 is changed to This study also found that the FR scale is sometimes used as an indicator of response postural balance ability

Line 192 was added: Different styles of Tai Chi interventions may improve postural balance ability. In this study, five items were Yang-style Tai Chi, and two items were Sun-style Tai Chi.

2. Gait: Line 201 added: Parkinson's patient measurements include four using walking velocity [15,41,42,48], three using stride length [15,41,48], and four using step length [15,35,42,48]. Two Parkinson's population measurements use tandem stance tests [40,44]. The gait measurement of the no-exercise population is mainly to compare gait velocity, step length, stride time, and cadence before and after. The mild MCI population also focuses on gait indicators the s but does not specify the specific measurement indicators.

3. Mobility: Line 208 is changed to: The findings showed that two of the three items in the Parkinson's group were functional mobility [40,49], which measured backward walking, 10-m walking, and TUG. One in the chronic stroke group, one in the MCI group using TUG measurements,

4. UPDRSⅢ：Line 216 added: It is worth noting that four of the eight articles pointed out that Yang-style Tai Chi intervention was used, but the impact on the results was different. Two studies showed that Yang-style Tai Chi intervention can improve UPDRSⅢ [40,41]. In contrast, UPDRSⅢ was not significant after the other two Yang-style Tai Chi interventions, which may be related to the different intervention cycles, frequencies, and time per week [47,48]. Other interventions on UPDRS III did not specify which style of Tai Chi was used.

5. Strength: Line 224 is changed: Two studies measure muscle strength in Fear of falls/history of falls and non-exercise populations

Line 224 is changed: Both studies focused on lower body muscle strength. These studies assessed lower body muscle strength using the 30-second chair stand

Line 227 added: Two studies used different styles of tai chi interventions, one with Sun tai chi intervention and the other with Yang style tai chi but the final intervention effect of both types of tai chi was significant.

6. Fall rate: Modify line 224: Two articles reported rates of falls were assessed in Parkinson's and Inactive populations, respectively [15,45]. One showed that the tai chi group had 67% fewer falls than the stretching group (incidence-rate ratio, 0.33; 95% CI, 0.16 to 0.71) [15]. Another study is not only about the rate of falls but also the risk of falls, the reduction of fear of falls has an improved effect [45].

Line 227: One of the Tai Chi intervention styles is Yang style intervention and the other is not mentioned.

7. QoL: Modify line 230: No mention was made of the quality of life of other groups of people.

Line 231 added: (median = 2.0 (25%: 2.0, 75%: 3.0)

Line 233 added: (7.91±1.81, 5.82±3.37, p=0.119)

Line 234 added: In terms of intervention style, one of them used Yang style intervention and the final result was effective., and the other did not mention Tai Chi style.

6. The results: in relation to TUG you have inclued in all measurements

TUG is funcional mobility- general mobility

you included in postural balance, gait, mobility dont think this is correct

**Response:** Lines 184-185 are about TUG deletion.

Line 201 is about TUG deletion.

7. line 219: Two studies evaluated strength in the elderly's fear of falls/history of falls and no exercise
I mentioned before fear isnt falls

what tipe of strength has been evaluated....

**Response:** Line 219: Two studies measure muscle strength in Fear of falls/history of falls and non-exercise populations. Both studies focused on lower body muscle strength. These studies assessed lower body muscle strength using the 30-second chair stand

Line 222: Two studies used different styles of tai chi interventions, one with Sun tai chi intervention and the other with Yang style tai chi but the final intervention effect of both types of tai chi was significant.

8. 236 needs the objectives before

**Response:** Line 236, Gait and balance problems is changed to gait and balance impairments.

9. 249 explain WHY did not improve

**Response:** Revise line 249: two gait indicators are Yang-style Tai Chi intervention for 16 weeks, of which 3 times a week for 1 hour can improve the gait of Parkinson's patients, while the other intervention is 2 times a week, every day Times 1 hour have no noticeable effect in people with Parkinson's disease.

10. 251-243 defined measures, explain as I mentioned before

Line 251-252 has been redefined to remove TUG.

11. 264 Why no improvment, explain

**Response:** This is a combined intervention for people with mild cognitive impairment. The results of the study showed that Taoist Tai Chi combined with MIP intervention did not have a significant effect.

12. 278 explain

**Response:** which may be due to multimodal exercise training in the control group the intervention effect is better than Tai Chi.

13. 300 why strength

**Response:** Decreased postural stability can lead to lower extremity impairment, and there is a strong link between lower extremity function and balance

14. 309 e 310 is not clear about rate of falls, explain and add the numbers

**Response:** lines 309-310 are modified: and one study showed that the tai chi group had a 55% rate of falls after the intervention compared to 67% in the stretching group, so the tai chi group was more effective.

15. 314-321 end the paragraph say about the relation of the title of the paper

**Response:** Lines 314-321 added: The results of this systematic review revealed the effects of different styles of Tai Chi intervention on improving postural balance, gait, mobility, UPDRS III, lower extremity muscle strength, and quality of life. Compared with the control group, most studies confirmed the positive effect of Tai Chi intervention on patients with gait disorders.

16. 340 you did not analyze (is meta-analyses) and rate of falls

**Response:** Lines 340: Nevertheless, there are few analyzes of falls in people with gait disorders in this article, and only two mentions are related to falls.

17. 350 say numbers frequency, weeks is better to improve

**Response:** Lines 350: high-frequency Tai Chi training (12 weeks, three times per week), and long-cycle low-frequency training (24 weeks, twice per week) could improve motor function in Parkinson's patients.

Reviewer #2:

1. - Line 13-31, abstract: Authors should report in full the content of the acronym when it first appears in the abstract.

**Response:** Line 16: Based on literature search principles, the following databases were searched:

Line 20: mild cognitive impairment (MCI), line 21: Parkinson's disease (PD), line 24: the Unified Parkinson's Disease Rating Scale Motor Subscale 3 (UPDRS III).

2. - Lines 27-31, the conclusions of the abstract should be more consistent with the conclusions of the work and above all report the limitations observed in all the works including the comparability of the studies, including duration, frequency of intervention, and type of movements, style.

**Response:** line 27-31: Tai Chi intervention benefits postural balance in patients with gait disorders. 12 weeks is the most common intervention period for patients with gait disorders. The frequency of intervention is seven articles twice a week, and the intervention time is about 60 minutes. The Tai Chi intervention methods in this study involve Yang Style, Sun Style, Taoist Tai Chi, and Health Qigong Tai Chi, but the Yang Style Tai Chi intervention is the most widely used.

3. - Line 85, "Tai Chi as an intervention method" the authors should better clarify what types of interventions are considered with the term Tai Chi and what styles are included.

**Response:** line 85: including all types of Tai Chi.

4. - Line 103, authors should consider to include a PRISMA Statement Checklist or similar as supplementary material

**Response:** Line 103 inserts a reference. [Fig 1]

Journal Requirements:

1. **Response:** Supporting information is added to line 364:

S1 Table. Detailed search strategy

S2 Table. PRISMA 2009 checklist

2. **Response:**

The date of the most recent literature review search has been added in the Methods section.
